# Underdetermined Wideband DOA Estimation for Off-Grid Sources with Coprime Array Using Sparse Bayesian Learning

**DOI:** 10.3390/s18010253

**Published:** 2018-01-16

**Authors:** Yanhua Qin, Yumin Liu, Jianyi Liu, Zhongyuan Yu

**Affiliations:** 1 Institute of Information Photonics and Optical Communications, Beijing University of Posts and Telecommunications, Beijing 100876, China; yanhuaqin@bupt.edu.cn (Y.Q.); yuzhongyuan30@hotmail.com (Z.Y.); 2 School of Cyberspace Security, Beijing University of Posts and Telecommunications, Beijing 100876, China; liujy@bupt.edu.cn

**Keywords:** coprime array, direction of arrival estimation, degrees of freedom, Sparse Bayesian learning, sparse signal representation, off-grid sources

## Abstract

Sparse Bayesian learning (SBL) is applied to the coprime array for underdetermined wideband direction of arrival (DOA) estimation. Using the augmented covariance matrix, the coprime array can achieve a higher number of degrees of freedom (DOFs) to resolve more sources than the number of physical sensors. The sparse-based DOA estimation can deteriorate the detection and estimation performance because the sources may be off the search grid no matter how fine the grid is. This dictionary mismatch problem can be well resolved by the SBL using fixed point updates. The SBL can automatically choose sparsity and approximately resolve the non-convex optimizaton problem. Numerical simulations are conducted to validate the effectiveness of the underdetermined wideband DOA estimation via SBL based on coprime array. It is clear that SBL can obtain good performance in detection and estimation compared to least absolute shrinkage and selection operator (LASSO), simultaneous orthogonal matching pursuit least squares (SOMP-LS) , simultaneous orthogonal matching pursuit total least squares (SOMP-TLS) and off-grid sparse Bayesian inference (OGSBI).

## 1. Introduction

Wideband direction of arrival (DOA) estimation using sensor arrays is an active research topic since it has broad applications requiring estimation the so-called angular spectrum, for example, in radar, sonar, wireless communication and localization, to name a few [1]. Because the DOA estimation accuracy is determined by the degrees of freedom (DOFs) of the sensor array, uniform spaced arrays need to increase the number of sensors to attain the high number of DOFs, and then raise the manufacturing cost as well as the difficulty of array calibration. Sparse arrays, i.e., nested array and coprime array [2,3], can obtain the higher number of DOFs to resolve more sources than the number of physical sensors using nonuniform sensor positions. Furthermore, for the sparse arrays the increased number of DOFs is achieved by exploiting the extended difference coarray, whose virtual sensor positions are determined by the consecutive and non-consecutive lag differences among the physical sensors.

Among sparse arrays, the coprime array has attracted considerable interest to the application of DOA estimation [4,5,6,7] because of the simplicity of the array configuration and the ability to detect more signals than the number of physical sensors. In [8], utilizing multiple frequencies to fill the missing coarray elements, the coprime array can effectively attain all of the offered DOFs for high resolution DOA estimation. Exploiting the sparse sparsity in the array signal model, the techniques of sparse signal representation [9,10,11] facilitate the progress of DOA estimation. These sparse signal representation based algorithms not only discretize the range of interest into a grid of spatial angles, but also assume that the true signal DOAs must fall on the predefined grid. However, in practical situations no matter how dense the grid is, the true DOAs may not necessarily lie on the exact sampling grid. This off-grid sources can cause the dictionary mismatch problem which not only violates the sparsity conditions but also deteriorates the performance [12]. In [13], the underdetermined wideband DOA estimation using group sparsity of two-step approach for off-grid sources is proposed to provide improved performance over the existing group sparsity based methods with the same search grid. In order to solve the problem that joint sparsity fails to capture the true structure of the signals, a novel wideband DOA estimation algorithm within the sparse Bayesian framework is proposed to allow a much more flexible occupation of the spectrum band, and automatically determine the underlying band occupation by imposing a Dirichlet process prior on the latent parametric space in [14].

Sparse Bayesian learning (SBL), as an alternative compressive sensing (CS) implementation, offers to relief the shortcoming that several sparse solutions might correspond to a single source when jointly processing multiple frequencies and multiple snapshots in order to localize one or more sources [15,16,17]. Being a probabilistic approach, SBL computes the posterior distribution of the sparse weight vectors and then provides estimations of their covariance along with the mean [18]. The idea of SBL applied to the single measurement vector (SMV) model for sparse signal recovery is to find the posterior probability $p\left(x|y;\Theta \right)$ via the Bayesian rule, where Θ indicates the set of all the hyperparameters [19,20,21]. The hyperparameters are estimated from data by marginalizing over *x* and then performing evidence maximization or Type-II Maximum likelihood [22]. The charm of SBL is that its global minima are always the sparest one [23], while the popular $l_{1}-norm$ based optimization algorithms [24] are not globally convergent. Therefore, the SBL based algorithms significantly outperform the traditional $l_{1}-norm$ based optimization algorithms.

In this paper, we focus on the underdetermined wideband DOA estimation for off-grid sources based on the coprime array using SBL algorithm. The coprime array, using minimal sparse rulers to reconstruct the spatial covariance matrix, enable nonuniform sampling approach which advocates the acquisition of a small number of samples to avoid aliasing for wideband signals. Vectorizing the covariance matrix and using kronecker product to the virtual manifold matrix from the coprime array, the DOA estimation for wideband signals can be obtained using the SBL algorithm. The SBL algorithm, developed within the sparse Bayesian framework, can approximately resolve the non-convex optimization problem and automatically determine sparsity using fixed point updates. The SBL scheme for wideband DOA estimation can provide processing advantages especially at low signal-to-noise ratio (SNR) under the acquisition of a small number of samples.

The remained of the paper is organized as follows. In Section 2 the signal model with coprime array is illustrated for wideband signals. In Section 3 the wideband DOA estimation of SBL for off-grid sources is introduced to the coprime array. No matter how fine the grid is, the true DOAs may be off the search grid. This off-grid problem can be well solved within sparse Bayesian framework. Section 4 presents numerical simulations showing that the DOA estimation for wideband signals using SBL has good performance in detection and estimation compared to least absolute shrinkage and selection operator (LASSO), SOMP-LS, SOMP-TLS and off-grid sparse Bayesian inference (OGSBI) based on coprime array. Section 5 concludes this paper.

## 2. Wideband Signal Model for Coprime Array

**Figure 1:**
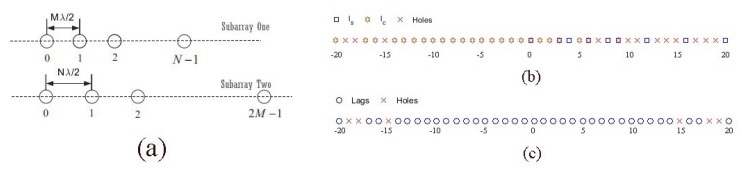
A coprime array configuration. (**a**) ULAs with sensor spacings related to a coprime array. (**b**) The sets $L_{s}$ and $L_{c}$ with M=3 and N=4. (**c**) The lags positions in full set $L_{p}$ with M=3 and N=4.

Consider a coprime array which is comprised of two uniform linear arrays (ULAs) with *N* and 2M sensors, as shown in Figure 1a, where the inter-element spacing of the first subarray is Mλ2 and that of the second subarray is Nλ2 with λ as the center wavelength of the signal. Assume that *K* far-field wideband sources $s_{k}\left(t\right),\;k=1,\ldots ,K$, impinge on the coprime array with incident angles $\Theta =\left[\theta_{1},\ldots ,\theta_{K}\right]$. Therefore, the signal observed at the *n*-th sensor of the coprime array can be expressed as

(1) $$x_{n}\left(t\right)=\sum_{k=1}^{k}s_{k}\left(t-\tau_{n}\left(\theta_{k}\right)\right)+{\bar{n}}_{n}\left(t\right)$$

where 0≤n≤2M+N−1, $s_{k}\left(t\right)$ is the kth signal, ${\bar{n}}_{n}\left(t\right)$ is the additive Gaussian white noise vector with the mean and variance equal to zero and $\sigma^{2}$ at the corresponding sensor and $\tau_{n}\left(\theta_{k}\right)$ represents the time delay of the *k*-th impinging signal with the angle $\theta_{k}$ arriving at the *n*-th sensor of the coprime array. Then the L-point discrete Fourier transform (DFT) is applied to the observed sensor signal and in frequency domain the data vector received at the *n*-th sensor can be expressed as

(2) $$X_{n}\left(l\right)=\sum_{i=0}^{L-1}x_{n}\left(i\right)e^{-j2\pi il/L}=\sum_{k=1}^{K}S_{k}\left(l\right)e^{-j2\pi f_{s}\tau_{n}\left(\theta_{k}\right)l/L}+{\bar{N}}_{n}\left(l\right)$$

where $S_{k}\left(l\right)=\sum_{i=1}^{L-1}s_{k}\left(t\right)e^{-j2\pi il/L}$ is the DFT of the *k*-th impinging signal $s_{k}\left(t\right)$ and ${\bar{N}}_{n}\left(l\right)$ is the DFT of the discrete time noise at *n*-th sensor of the coprime array. l=1,…,L,L≥K and $f_{s}$ denotes the sampling frequency. The output signal model in the DFT domain can be written into

(3) $$X\left(l\right)=A\left(l,\theta \right)S\left(l\right)+\bar{N}\left(l\right)$$

where $A\left(l,\theta \right)=\left[a\left(l,\theta_{1}\right),\ldots ,a\left(l,\theta_{K}\right)\right]$ is the steering matrix.

The covariance matrix of data vector can be obtained as

(4) $$R_{l}=E\left\{X\left(l\right)\cdot X^{H}\left(l\right)\right\}=\sum_{k=1}^{K}\sigma_{k}^{2}\left(l\right)a\left(l,\theta_{k}\right)a^{H}\left(l,\theta_{k}\right)+\sigma_{\bar{n}}^{2}\left(l\right)I_{2M+N-1}$$

where $E\left\{\cdot \right\}$ is the expection operator and ${\left\{\cdot \right\}}^{H}$ is the Hermitian transpose operator. $\sigma_{k}^{2}\left(l\right)$ denotes the power of the *k*-th impinging signal, while $\sigma_{\bar{n}}^{2}\left(l\right)$ denotes the corresponding noise power. In practical situations, the theoretical covariance matrix $R_{l}$ is unavailable and the sample covariance matrix ${\hat{R}}_{l}$ can be estimated using the *T* available segments (frequency snapshots) as

(5) $$R_{l}\approx {\hat{R}}_{l}=\frac{1}{T}\sum_{t=1}^{T}X\left(l,p\right)\cdot X^{H}\left(l,p\right)$$

Then vectorizing $R_{l}$ in column and using kronecker product to obtain the following virtual array model

(6) $$z_{l}=vec\left(R_{l}\right)=B_{l}u_{l}+\sigma_{\bar{n}}^{2}\left(l\right){\bar{I}}_{{\left(2M+N-1\right)}^{2}}$$

where $B_{l}=\left[b\left(l,\theta_{1}\right),\ldots ,b\left(l,\theta_{K}\right)\right]$ with $b\left(l,\theta_{k}\right)=a^{*}\left(l,\theta_{k}\right)⊗a\left(l,\theta_{k}\right)$, $u_{l}=\left[\sigma_{1}^{2}\left(l\right),\ldots ,\sigma_{K}^{2}\left(l\right)\right]$ and ${\bar{I}}_{{\left(2M+N-1\right)}^{2}}=vec\left(I_{2M+N-1}\right)$ in which the symbol ${}^{\prime }{*}^{\prime }$ denotes complex conjugation, the symbol ${}^{\prime }{⊗}^{\prime }$ denotes the Kronecker product and $vec\left(\cdot \right)$ denotes the vectorization operation.

The locations of the sensors from the matrix $B_{l}$, regarded as the manifold matrix of a larger virtual array, are in the self-difference set

$$L_{s}=\left\{l_{s}|l_{s}=Nm,\;1\leq m\leq 2M-1\right\}\cup \left\{l_{s}|l_{s}=Mn,0\leq n\leq N-1\right\}$$

cross-difference set

$$L_{c}=\left\{\left(Mn-Nm\right),\;0\leq n\leq N-1,\;1\leq m\leq 2M-1\right\}$$

the corresponding mirrored self-difference set $L_{s}^{-}=\left\{-l_{s}|l_{s}\in L_{s}\right\}$ and the corresponding mirrored cross-difference set $L_{c}^{-}=\left\{-l_{c}|l_{c}\in L_{c}\right\}$ [25]. Therefore, the full set of lags from the larger virtual array is $L_{p}=L_{s}\cup L_{s}^{-}\cup L_{c}\cup L_{c}^{-}$. As a example of the coprime array with M=3 and N=4, Figure 1b shows the self-difference and cross-difference sets, while Figure 1c illustrates the full set of lags. Using the full set of lags, the resulting coarray can offer the higher number of DOFs to resolve more sources than the number of physical sensors.

## 3. Sparse Bayesian Learning with Off-Grid Sources

### 3.1. Off-Grid Formulation

It is assumed that there are Q≤L DFT frequency bins indexed by $l_{q},\;q=1,\ldots ,Q$, which may or may not occupy the consecutive frequency bands within the bandwidth of signals. Then the manifold matrix $B_{l}$ corresponding to the *Q* frequency bins is $B_{l}=\left[B_{l_{1}},\ldots ,B_{l_{Q}}\right].$ Sampling the potential spatial domain with D-element grid $\theta^{g}$ with fixed spacing $r^{g}=\theta_{l+1}^{g}-\theta_{l}^{g},1\leq l\leq D$ and discretizing the grid interval as $\theta_{1}^{g},\theta_{2}^{g},\ldots ,\theta_{D}^{g},\;D\gg K$, the virtual array model (6) can be written into

(7) $$z_{l}=B_{l}^{o}u_{l}^{o}+\sigma_{\bar{n}}^{2}\left(l\right){\bar{I}}_{{\left(2M+N-1\right)}^{2}}$$

where $\;z_{l}=\left[z_{l_{1}},\ldots ,z_{l_{Q}}\right]$, $B_{l}^{o}=\left[b\left(l,\theta_{1}^{g}\right),\ldots ,b\left(l,\theta_{D}^{g}\right)\right]$ with $b\left(l,\theta_{l}^{g}\right)=a^{*}\left(l,\theta_{l}^{g}\right)⊗a\left(l,\theta_{l}^{g}\right)$ and $u_{l}^{o}={\left[\sigma_{1}^{2}\left(l\right),\ldots \;,\sigma_{D}^{2}\left(l\right)\right]}^{T}$ which is a sparse vector, whose elements are all zeros except those corresponding to the true DOAs. No matter how fine the grid is, the true DOAs $\theta_{k}$, denoted as $\theta_{k}=\theta_{l}^{g}+\Delta_{l}$ where $\theta_{l}^{g}\in \theta^{g}$ denotes the nearest grid corresponding to the $\theta_{k}$ and $\Delta_{l}\in \left[-\frac{r^{g}}{2}\;\;\frac{r^{g}}{2}\right]$ is the grid offset, may be off the dense search grid $\theta^{g}$. The steering vector $a\left(l,\theta_{l}^{g}+\Delta_{l}\right)$ at the actual angle $\theta_{k}$ can be approximately expressed as

$$a\left(l,\theta_{l}^{g}+\Delta_{l}\right)=a\left(l,\theta_{l}^{g}\right)⊙c\left(l,\Delta_{l}\right)$$

where $c\left(l,\Delta_{l}\right)={\left[1,e^{\frac{-j2\pi f_{l}M\lambda \cos\left(\Delta_{l}\right)}{2c}},\ldots ,e^{\frac{-j2\pi f_{l}N\lambda \left(2M-1\right)\cos\left(\Delta_{l}\right)}{2c}}\right]}^{T}.$ Then the equivalent steering vector $b\left(l,\theta_{l}^{g}+\Delta_{l}\right)$ at the actual angle $\theta_{k}$ can be expressed as

(8) $$\begin{array}{cc} b\left(l,\theta_{l}^{g}+\Delta_{l}\right) & =a^{*}\left(l,\theta_{l}^{g}+\Delta_{l}\right)⊗a\left(l,\theta_{l}^{g}+\Delta_{l}\right)\\ & ={\left(a\left(l,\theta_{l}^{g}\right)⊙c\left(l,\Delta_{l}\right)\right)}^{*}⊗\left(a\left(l,\theta_{l}^{g}\right)⊙c\left(l,{△}_{l}\right)\right)\\ & =\left(a^{*}\left(l,\theta_{l}^{g}\right)⊗a\left(l,\theta_{l}^{g}\right)\right)⊙\left(c^{*}\left(l,{△}_{l}\right)⊗c\left(l,{△}_{l}\right)\right) \end{array}$$

Denote the collection of D steering vectors as ${\tilde{B}}_{l}=\left[b\left(l,\theta_{1}^{g}+{△}_{1}\right),\ldots ,b\left(l,\theta_{D}^{g}+\Delta_{D}\right)\right]$, the off-grid virtual array model can be given as

$$z_{l}=\tilde{B_{l}}u_{l}^{o}+v_{l}$$

where $v_{l}$ denotes the vector comprising of all zeros except its *k*-th entry corresponding to the variance of the *k*-th element of the ${\bar{n}}_{n}\left(t\right)$.

### 3.2. Sparse Bayesian Learning Algorithm

Due to the noise being Gaussian, the likelihood can be expressed as

(9) $$p\left(z_{l_{q}}|u_{l_{q}}^{o};{\tilde{B}}_{l_{q}}\right)=\mathrm{CN}\left(z_{l_{q}}|{\tilde{B}}_{l_{q}}u_{l_{q}}^{o},\;\sigma_{\bar{n}}^{2}I\right)$$

Since $u_{l_{q}}^{o}$ is real and nonnegative, Equation (9) can be converted to be the following real-valued likelihood form

(10) $$p\left(y_{l_{q}}|u_{l_{q}}^{o};{\tilde{B}}_{l_{q}}\right)=\mathrm{CN}\left(y_{l_{q}}|{\tilde{B}}_{l_{q}}u_{l_{q}}^{o},\;\sigma_{\bar{n}}^{2}I\right)$$

where $y_{l_{q}}\overset{△}{=}{\left[Re\left(z_{l_{q}}^{T}\right),\;Im\left(z_{l_{q}}^{T}\right)\right]}^{T}$ and ${\tilde{B}}_{l_{q}}\overset{△}{=}{\left[Re\left({\tilde{B}}_{l_{q}}^{T}\right),\;Im\left({\tilde{B}}_{l_{q}}^{T}\right)\right]}^{T}$ [26]. Using the Gaussian distribution, the prior for $u_{l_{q}}^{o}$ can be expressed as

$$p\left(u_{l_{q}}^{o};\;\gamma_{l_{q}}\right)=\mathrm{CN}\left(u_{l_{q}}^{o}|0,\;\Gamma_{l_{q}}\right)=\mathrm{CN}\left(u_{l_{q}}^{o}|0,\;diag\left(\gamma_{l_{q}}\right)\right)=\prod_{m=1}^{D}\mathrm{CN}\left(u_{l_{q},m}^{o}|0,\;\gamma_{l_{q},m}\right)$$

where $\Gamma_{l_{q}}=diag\left(\gamma_{l_{q},1},\ldots ,\gamma_{l_{q},D}\right)=diag\left(\gamma_{l_{q}}\right)$ is the diagonal covariance of the source amplitudes with the vector $\gamma_{l_{q}}$ as the source power in each range-depth cell θ.

Let $Y_{l_{q}}=\left[y_{1_{q}},\ldots ,y_{L_{q}}\right]$ denote the collection of *T* snapshots and the corresponding collection of source variance vectors be denoted as $U_{l_{q}}^{o}$, respectively. Using Equation (10), the multi snapshot likelihood can be expressed as

$$p\left(Y_{l_{q}}|U_{l_{q}}^{o}\right)=\prod_{t=1}^{T}p\left(y_{l_{q},t}|u_{l_{q},t}^{o}\right)$$

The multi frequency likelihood can be expressed as

$$p\left(Y_{l_{1:Q}}|U_{l_{1:Q}}^{o}\right)=\prod_{q=1}^{Q}\prod_{t=1}^{T}p\left(y_{l_{q},t}|u_{l_{q},t}^{o}\right)$$

The evidence $p\left(Y_{l_{q}}\right)$ can be obtained by averaging over all realizations of $U_{l_{q}}^{o}$ as

(11) $$\begin{array}{cc} p\left(Y_{l_{q}}\right) & =\int p\left(Y_{l_{q}}|U_{l_{q}}^{o}\right)p\left(U_{l_{q}}^{o}\right)dU_{l_{q}}^{o}=\int \prod_{t=1}^{T}\mathrm{CN}\left(y_{l_{q},t};{\tilde{B}}_{l_{q}}u_{l_{q},t}^{o},\;\sigma_{\bar{n}}^{2}I\right)\mathrm{CN}\left(u_{l_{q},t}^{o};0,\;\Gamma_{l_{q}}\right)\\ & =\prod_{t=1}^{T}\mathrm{CN}\left(y_{l_{q},t};0,\sigma_{\bar{n}}^{2}I+{\tilde{B}}_{l_{q}}\Gamma_{l_{q}}{\tilde{B}}_{l_{q}}^{H}\right)=\prod_{t=1}^{T}\mathrm{CN}\left(y_{l_{q},t};0,\sum_{y_{l_{q}}}\right) \end{array}$$

where $\sum_{y_{l_{q}}}=\sigma_{\bar{n}}^{2}I+{\tilde{B}}_{l_{q}}\Gamma_{l_{q}}{\tilde{B}}_{l_{q}}^{H}$. To estimate $\gamma_{l_{q}}$ which denotes $\hat{\gamma_{l_{q}}}$, we maximize the joint evidence

$${\hat{\gamma }}_{l_{1:Q}}=\underset{\gamma_{l_{1:Q}}}{\arg\max\;p\left(Y_{l_{1:Q}}\right)=}\underset{\gamma_{l_{1:Q}}}{\arg\min}\left\{\sum_{q=1}^{Q}T\log|\sum_{y_{l_{q}}}|+Tr\left(Y_{l_{q}}^{H}\sum_{y_{l_{q}}}^{-1}Y_{l_{q}}\right)\right\}$$

where Tr() denotes the trace of a matrix and ∣·∣ denotes the determinant of a matrix. To get the minimum of this objection function, we equate the derivative of the objection function to zero

$$\frac{\partial }{\partial \gamma_{l_{q},m}}\left\{\sum_{q=1}^{Q}T\log|\sum_{y_{l_{q}}}|+Tr\left(Y_{l_{q}}^{H}\sum_{y_{l_{q}}}^{-1}Y_{l_{q}}\right)\right\}=0$$

(12) $${\hat{\gamma }}_{m}^{new}={\hat{\gamma }}_{m}^{old}\frac{\sum_{q=1}^{Q}∥Y_{l_{q}}^{H}\sum_{y_{l_{q}}}^{-1}\tilde{b}\left(l_{q},m\right){∥}_{2}^{2}}{T\sum_{q=1}^{Q}{\tilde{b}}^{H}\left(l_{q},m\right)\sum_{y_{l_{q}}}^{-1}\tilde{b}\left(l_{q},m\right)}$$

where $\tilde{b}\left(l_{q},m\right)={\left[Re\left(b^{T}\left(l_{q},m\right)\right),\;Im\left(b^{T}\left(l_{q},m\right)\right)\right]}^{T}$. If the sparsity is the same with different frequencies, the estimate of $\hat{\gamma_{l_{q}}}$ correspond to the source variance at the q-th frequency bin. Denote ${\tilde{B}}_{M}$ the matrix formed by K columns of $\tilde{B}$ indexed by M, where the set M signifies the location of the non-zero entries of γ with cardinality ∣M∣=K. The variance of noise $\sigma_{\bar{n}}^{2}$ can be expressed as

(13) $$\sigma_{\bar{n}}^{2}=\frac{1}{D-K}Tr\left(\left(I_{D}-{\tilde{B}}_{l,M}{\tilde{B}}_{l,M}^{+}\right){\tilde{R}}_{l}\right)$$

where ${\tilde{B}}_{M}^{+}$ denotes the Moore-Penrose pseudo-inverse of the matrix ${\tilde{B}}_{M}$ and ${\tilde{R}}_{l}=R_{l}^{T}⊗R_{l}/T$ [17].

In short, the SBL algorithm can be summarized as follows:

Step 1. Initialization: $\epsilon =10^{-4}$, $\gamma_{m}^{old}=1$, $\sigma_{\bar{n}}^{2}=0.1$, $N_{t}=1$
Step 2. Input $y_{l}$ with $y_{l}=\left[y_{l_{1}},\ldots ,y_{l_{Q}}\right]$ and ${\tilde{B}}_{l}$ with ${\tilde{B}}_{l}=\left[{\tilde{B}}_{l_{1}},\ldots ,{\tilde{B}}_{l_{Q}}\right]$, then compute $\sum_{y_{l}}=\sigma_{\bar{n}}^{2}I+{\tilde{B}}_{l}\Gamma_{l}{\tilde{B}}_{l}^{H}$
Step 3. Update $\gamma_{m}^{new}$ according to Equation (12)
Step 4. Update $\sigma_{\bar{n}}^{2}$ according to Equation (13)
Step 5. If $\frac{∥\gamma^{new}-\gamma^{old}{∥}_{1}}{∥\gamma^{new}{∥}_{1}}<\epsilon$, stop
Step 6. let $\gamma^{old}=\gamma^{new}$ and $N_{t}=N_{t}+1$
Step 7. If $N_{t}<1000$, go to step 2; otherwise stop.

## 4. Simulation Result

In this section, we carry out simulations to illustrate the performance of SBL with the coprime array for wideband DOA estimation, and also compare it with other state-of-the-art algorithms, including LASSO, SOMP-LS, SOMP-TLS [27] and OGSBI [28]. In the simulations, the fractional bandwidth, which is the ratio of bandwidth divided by the center frequency, is 2/3 for signals. The sampling frequency is three times the highest frequency. In other words, the signals have a normalized frequency range from (1/3)π to (2/3)π, where the normalized frequency can be defined as $\omega =\frac{2f}{f_{s}}\pi$ with *f* being the frequency of interest. Considering the signals at each frequency bin sharing the same distribution, i.e., the amplitude being a Rayleigh random variable and the phase being uniformly distributed on $\left[-\pi ,\pi \right]$. L=128-point DFT is applied and the frequency band of interest covers Q=26 frequency bins. Assume that an example of K=12 signals with their off-grid impinging angles uniformly distributed between −π3 and π3. The coprime array consists of a pair of sparse ULAs with M=3 and N=4, and in total there are 9 physical sensors considered with the position set S=0,3,4,6,8,9,12,16,20λ2. A search grid of $K_{g}=\frac{2\pi }{r}$ potential angles corresponding to a step size r=1 is generated with the full angle range from $-\frac{\pi }{2}$ to $\frac{\pi }{2}$.

**Figure 2:**
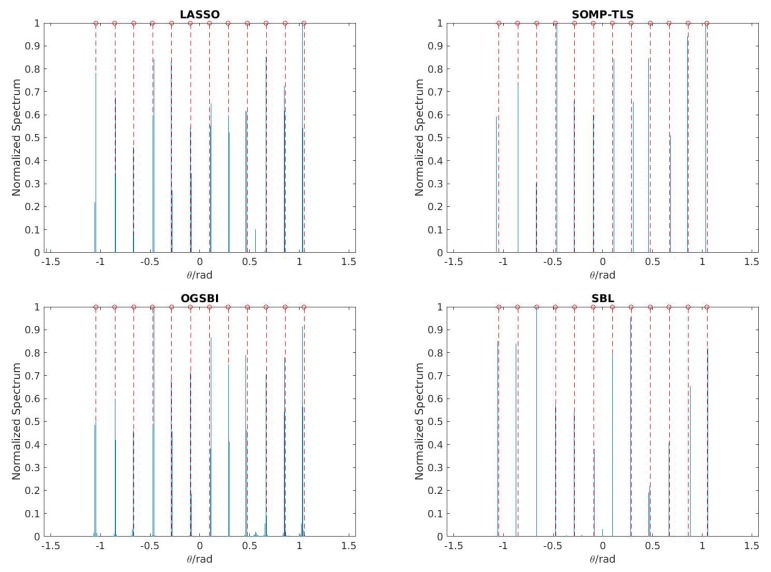
Normalized spectra for least absolute shrinkage and selection operator (LASSO), simultaneous orthogonal matching pursuit total least squares (SOMP-TLS), off-grid sparse Bayesian inference (OGSBI) and Sparse Bayesian learning (SBL) with T=100 and signal-to-noise ratio (SNR) =0 dB.

First, we compare the detection performance of SBL with that of LASSO, SOMP-TLS and OGSBI. Within the entire frequency band of interest the signal power and the noise power are used to calculate the SNR. Assume that K=12 wideband signals impinge on the coprime array with M=3 and N=4, the number of snapshots is 100 and the input SNR is fixed to be 0 dB. In Figure 2, the solid lines represent the estimation of DOAs, while the dotted lines represent the actual incident angles of source signals. As shown in Figure 2, all wideband signals (more than the number of physical sensors) can be distinguished successfully by LASSO, SOMP-TLS, OGSBI and SBL. However, LASSO and OGSBI may generate some spurious peaks for sources. There is no spurious peaks with the performance of SOMP-TLS compared to the other methods, even SBL has one spurious peak at 0.5 rad. Also, for some angles SOMP-TLS provides closer DOA estimates to the true values compared to SBL, whereas SBL gives better accuracy for the other angles. Therefore, the estimation performance using SBL and SOMP-TLS methods are comparable and provide the best detection among the other methods.

**Figure 3:**
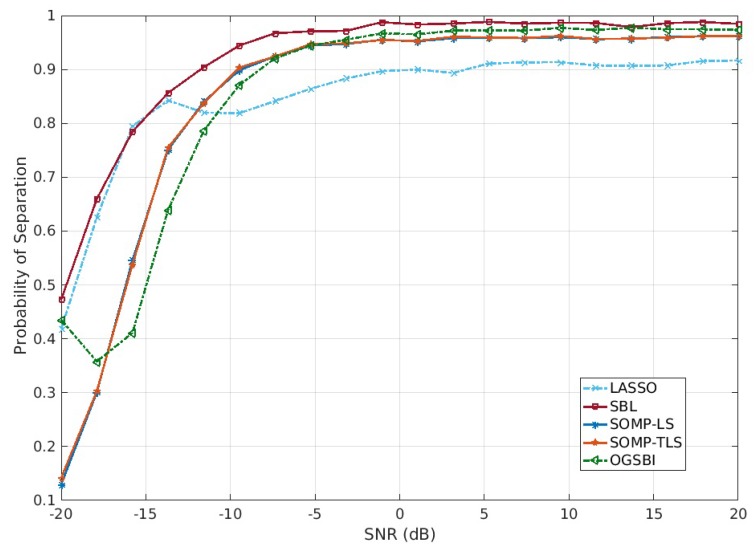
Separation probabilities vs. SNR with T=200 based on coprime array.

Next, we compare the separation ability of SBL with that of LASSO, SOMP-LS, SOMP-TLS and OGSBI in the statistical sense for SNR. For each angle separation of the signals, the separation ability of all algorithms are derived from 200 trials. Assume Δθ=0.0952 rad as half of the two closely incident signals, successful separation of LASSO, SOMP-LS, SOMP-TLS, OGSBI and SBL is defined if the estimated DOA of each signal satisfies $\theta_{k}-\Delta \theta \leq \hat{\theta_{k}}\leq \theta_{k}+\Delta \theta$. In other words, we carry out the criteria, the DOA estimation biases of each signal do not exceed Δθ, for separation evaluation. Assume that K=12 wideband signals as in the first experiment impinge on the coprime array with M=3 and N=4, the number of snapshots is fixed to be 200 and the SNR is varied from −20 dB to 20 dB for each trial. Figure 3 illustrates the separation performance based on the coprime array for LASSO, SOMP-LS, SOMP-TLS and OGSBI. It is evident that SBL performs best compared to LASSO, SOMP-LS, SOMP-TLS and OGSBI.

Finally, the estimation performance of SBL is conducted to evaluate the estimation accuracy in comparison with that of LASSO, SOMP-LS, SOMP-TLS and OGSBI in terms of the root mean square error (RMSE). The empirical RMSE of the estimated DOAs, defined as $RMSE=\sqrt{\sum_{i=1}^{W}\sum_{k=1}^{k}{\left({\hat{\theta }}_{k}(i)-\theta_{k}\right)}^{2}}$, where W is the number of independent Monte Carlo trials and ${\hat{\theta }}_{k}\left(i\right)$ is the estimate of $\theta_{k}$ in the *i*th Monte Carlo trial, is used to evaluate the simulation performance. Here each testing point is based on an average of results by 200 iterations of Monte Carlo simulations. Assume that K=12 wideband signals as in the first experiment impinge on the coprime array with M=3 and N=4 for each iteration. Crame-Rao lower bound (CRLB) [29,30,31,32,33], which offers a lower bound on the variances of estimation accuracy, is also used to indicate the ideal estimation.

**Figure 4:**
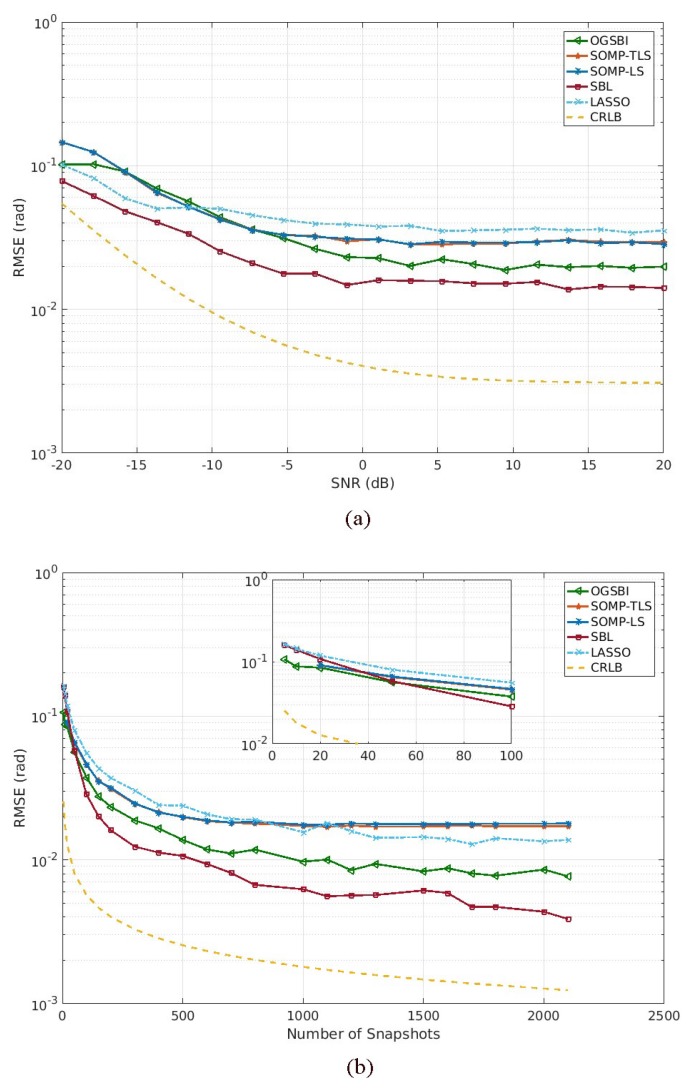
Estimation accuracy for 12 wideband signals based on coprime array. (**a**) RMSE vs. input SNR with T=200 snapshots. (**b**) RMSE vs. the number of snapshots with SNR=0 dB.

Figure 4a depicts the RMSE of LASSO, SOMP-LS, SOMP-TLS, OGSBI and SBL versus SNR for wideband signals based on coprime array with the number of snapshots T=200. It is clear that the DOA estimation performance is improved with the increase of SNR for all algorithms. Note that the RMSE of the SBL is the lowest compared to that of LASSO, SOMP-LS, SOMP-TLS and OGSBI. Therefore, the performance of SBL outperforms LASSO, SOMP-LS, SOMP-TLS and OGSBI when SNR is varied from −20 dB to 20 dB. The reason is that the all-on-grid assumption severely degrades the performance of LASSO, but SBL can efficiently alleviate the off-grid mismatch problem.

Figure 4b plots the RMSE of LASSO, SOMP-LS, SOMP-TLS, OGSBI and SBL versus the number of snapshots for wideband signals based on coprime array with SNR=0 dB. Due to the rate of successful detection using SOMP-LS and SOMP-TLS be 27% when T=10, their RMSE values are considered from T=20. It can be readily observed that the estimation performance is to be increased as the number of snapshots is increasing for all algorithms. As shown in Figure 4b, SBL can obtain more accurate estimation performance by increasing the number of snapshots in comparison with LASSO, SOMP-LS, SOMP-TLS and OGSBI. Therefore, the superiority of the off-grid mismatch for SBL is demonstrated.

## 5. Conclusions

In this paper, by exploiting SBL for the underdetermined DOA estimation of wideband signals based on the coprime array, we find that the SBL can achieve superior detection performance and estimation accuracy in comparison to LASSO, SOMP-LS, SOMP-TLS and OGSBI. The SBL can accommodate the increased DOFs provided by the coprime array to perform the effective underdetermined wideband DOA estimation for off-grid sources. The SBL employs fixed point updates to give global convergence properities for wideband DOA estimation. Numerical experiments are used to demonstrate the superiority of the SBL in detection and estimation performance with the coprime array for underdetermined wideband DOA estimation.
